# Highly Integrated All-Fiber FP/FBG Sensor for Accurate Measurement of Strain under High Temperature

**DOI:** 10.3390/ma11101867

**Published:** 2018-10-01

**Authors:** Tingting Yang, Xiu He, Zengling Ran, Zhendong Xie, Yunjiang Rao, Xueguang Qiao, Zhengxi He, Peng He

**Affiliations:** 1 Fiber Optics Research Center, Key Laboratory of Optical Fiber Sensing & Communications (Ministry of Education), University of Electronic Science & Technology of China, Chengdu 611731, China; ttyang@stumail.nwu.edu.cn (T.Y.); 18883242676@163.com (X.H.); wsdewqa@126.com (Z.X.); yjrao@uestc.edu.cn (Y.R.); 2 Department of Physics, Northwest University, Taibai Beilu 229, Xi’an 710069, China; xgqiao@nwu.edu.cn; 3 Nuclear Power Institute of China, Chengdu 610041, China; hezhengxi0002@163.com (Z.H.); 15202899481@163.com (P.H.)

**Keywords:** optical fiber sensors, strain/pressure sensing, Fabry–Perot, FBG, dual-parameter measurement, high temperature

## Abstract

Accurate measurement of strain is one of the most important issues for high temperature environments. We present a highly integrated all-fiber sensor to achieve precise measurements of strain/high-pressure, which consists of a fiber Bragg grating (FBG) inscribed by an 800 nm femtosecond laser cascaded with a micro extrinsic Fabry–Perot (FP) cavity fabricated by the 157 nm laser micromachining technique. FBG is sensitive to temperature, but insensitive to strain/pressure, whereas the FP is sensitive to strain/pressure, but has a small dependence on temperature. Therefore, such a cascaded sensor could be used for dual-parameter measurement and can work well at high temperatures. Experimental results indicate that this device exhibits a good strain characteristic at high temperatures and excellent high-pressure performance at room temperature. Due to its highly sensitive wavelength response, the proposed sensor will have remarkable potential applications in dual parameter sensing in harsh environments.

## 1. Introduction

Accurate measurement of strain under high temperatures is one of the most important issues for many industrial applications, such as downhole and aero-engine monitoring, etc. [1,2,3]. However, it is quite challenging to achieve accurate measurement of strain under high temperatures as the thermal-induced drift of the sensor would be significant and hence cause a relatively large measurement error under high temperatures. Simultaneous measurement of temperature and strain is an effective method to solve the cross-sensitivity problem in fiber-optic sensors. Over the decades, a variety of approaches based on different mechanisms have been proposed and demonstrated for simultaneous measurement of temperature and strain, including a pair of regenerated gratings (RGs) [4], a Mach–Zehnder interferometer based on a Z-Shape Fiber Structure [5], two Fabry–Perot (FP) cavities [6], and cascaded fiber Bragg gratings (FBGs) with different sensitivity coefficients [7]. To achieve a higher accuracy for multi-parameter measurement, a highly integrated sensor head that can perform the functions for simultaneous measurement of dual parameters is much more desirable for high-temperature applications. As a typical fiber-optic sensor, FBG performs well in temperature response, while FP has good sensitivity to strain due to its unique micro air cavity structure inside the fiber with tiny thermal drift. Therefore, combining FBG and FP can greatly improve the accuracy of fiber-optic strain sensors via compensation of the thermal induced drift. So far, many configurations based on hybrid fiber gratings and an FP interferometer (FPI) have been reported for simultaneous strain and temperature, such as a fiber grating cascaded with an FP [8,9,10], and a fiber grating overlapped on an FP cavity [11,12,13]. Among these sensors, FBGs are inscribed by an ultraviolet (UV) laser, which has low thermal stability under high temperatures, and regenerated FBG has a low mechanical strength. Thus, the performance of the sensors still needs to be further improved. In recent years, a femtosecond laser has been used to write FBG, and has drawn increasing attention due to its unique advantages [14]. Jiang et al. [15] implemented a sensor, which consists of a capillary-based FP interferometer and an FBG inscribed by a femtosecond laser, for strain (0–1122 με) and temperature (23 °C–600 °C) discrimination. However, the sensor is capillary-based. Therefore, there is a strong desire for developing a high performance all-fiber sensor that can measure strain accurately in high-temperature environments.

Here, a novel all-fiber in-line sensor cascading a short FBG written by a femtosecond laser and a micro air cavity FP fabricated by a 157 nm laser is proposed and demonstrated. The 157 nm laser is ideal to be used to achieve high quality micromachining due to it has high pulse energy and single-photon and a high absorption coefficient for many materials. The FBG is arranged to be very close to the FP to avoid any measurement error caused by temperature/measurand difference at different positions. The FBG is sensitive to temperature, but insensitive to strain/pressure. The FP is sensitive to strain/pressure, but insensitive to temperature. The integrated FP/FBG sensor shows excellent characteristics for accurate measurement of strain under high temperatures of up to 500 °C and high-pressures of up to 23 MPa at room temperature. Additionally, the FBG and the FP, fabricated using laser micromachining technology, can not only work well at high temperatures, but also have good consistency for mass production, making them quite promising to be used in high temperature, harsh environments.

## 2. Operating Principle

The sensing principle of the FP/FBG sensor for simultaneous measurement of strain and temperature is based on the sensing properties of the FBG and FP cavity, respectively. The FBG is sensitive to temperature for the silica fiber has a high thermo-optic coefficient (≈10 ppm/°C) [6] and is insensitive to strain. Hence, it is mainly used to measure temperature. However, the FP is sensitive to strain, while it has a small temperature dependence due to the air in the FP cavity, which has a low thermo-optic coefficient (≈0.1 ppm/°C) [6]; therefore, the FP is mainly used to measure strain based on the change in cavity length when strain is applied to the sensor. Eventually, the change of wavelength with temperature and strain can be expressed as:

(1) $$\Delta \lambda_{1}=\alpha_{1}\cdot \Delta T+\beta_{\varepsilon 1}\cdot \Delta \varepsilon ,$$

(2) $$\Delta \lambda_{2}=\alpha_{2}\cdot \Delta T+\beta_{\varepsilon 2}\cdot \Delta \varepsilon ,$$

where Δ*λ*_1_ and Δ*λ*_2_ are the resonance wavelengths’ shift of the FBG and the FP, respectively. Δ*T* and Δ*ε* are the change in temperature and strain, respectively. In addition, *α*_1_ and *α*_2_ represent the temperature sensitivities of the FBG and the FP, respectively. The strain responses of the FBG and the FP are *β_ε_*_1_ and *β_ε_*_2_, respectively. Once those response coefficients of both the temperature and strain are obtained, Δ*T* and Δ*ε* can be expressed using Equations (1) and (2):

(3) $$\Delta T=\frac{\beta_{\varepsilon 2}\cdot \Delta \lambda_{1}-\beta_{\varepsilon 1}\cdot \Delta \lambda_{2}}{\alpha_{1}\cdot \beta_{\varepsilon 2}-\alpha_{2}\cdot \beta_{\varepsilon 1}},$$

(4) $$\Delta \varepsilon =\frac{\alpha_{2}\cdot \Delta \lambda_{1}-\alpha_{1}\cdot \Delta \lambda_{2}}{\alpha_{2}\cdot \beta_{\varepsilon 1}-\alpha_{1}\cdot \beta_{\varepsilon 2}},$$

Based on the different sensing coefficients of the FBG and FP cavity, the sensor can be used for simultaneous measurement of the pressure and temperature. The FBG is sensitive to temperature while insensitive to pressure. Similarly, it is mainly used to measure temperature. Besides, pressure measurement is achieved using the FP based on the change in cavity length when the pressure is applied to the fiber. The resonance wavelengths’ change of the FBG and FP with temperature and pressure can be expressed as:

(5) $$\Delta \lambda_{1}=\alpha_{1}\cdot \Delta T+\beta_{P1}\cdot \Delta P,$$

(6) $$\Delta \lambda_{2}=\alpha_{2}\cdot \Delta T+\beta_{P2}\cdot \Delta P,$$

where Δ*T* and Δ*P* are the change in temperature and pressure, respectively. The pressure responses of the FBG and the FP are *β_P_*_1_ and *β_P_*_2_, respectively. Similarly, Δ*T* and Δ*P* can be expressed as follows using Equations (5) and (6):

(7) $$\Delta T=\frac{\beta_{P2}\cdot \Delta \lambda_{1}-\beta_{P1}\cdot \Delta \lambda_{2}}{\alpha_{1}\cdot \beta_{P2}-\alpha_{2}\cdot \beta_{P1}},$$

(8) $$\Delta P=\frac{\alpha_{2}\cdot \Delta \lambda_{1}-\alpha_{1}\cdot \Delta \lambda_{2}}{\alpha_{2}\cdot \beta_{P1}-\alpha_{1}\cdot \beta_{P2}},$$

## 3. Fabrication of the Sensor

**Figure 1:**
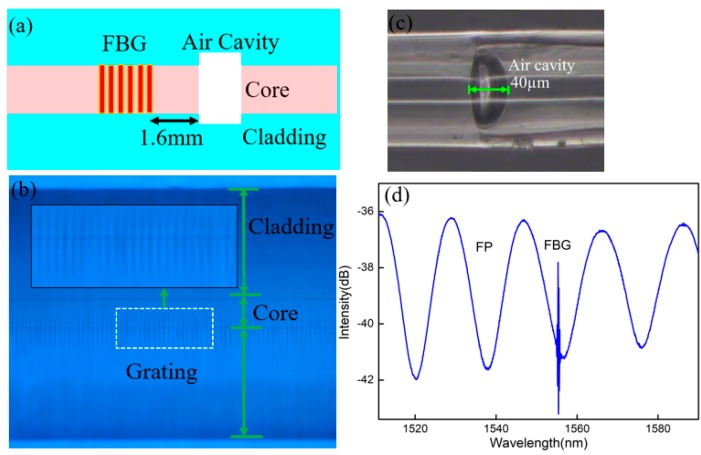
(**a**) Schematic diagram of the sensor head; (**b**) Micrograph of the FBG; (**c**) micrograph of the FP; (**d**) mixed spectrum of the FBG and the FP cavity.

**Figure 2:**
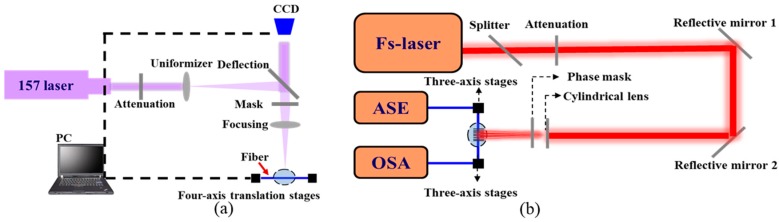
Schematic of the fabrication system. (**a**) 157 nm laser micromachining system; (**b**) 800 nm femtosecond laser system.

The schematic diagram of the sensor consisting of an FBG and an FP cavity is shown in Figure 1a. Additionally, the FP is only 1.6 mm away from the FBG. Firstly, a 157 nm micromachining system is used to form a micro-hole in the core of a cleaved fiber end-face. The micromachining system is similar to that used in reference [3], as shown in Figure 2a. The pulse repetition rate and pulse energy density are 20 Hz and 20 J/cm^2^, respectively. Subsequently, the machined fiber is spliced with another cleaved single mode fiber (SMF) to form an FP cavity of ~40 μm in length. Then, a Ti:sapphire femtosecond laser with a repetition rate of 1 kHz and a pulse duration of 50 fs is used to write an FBG. The femtosecond laser system is similar to that used in reference [16], as shown in Figure 2b. A phase mask (produced by Ibsen Photonics) with a grating pitch of 2142 nm and the first order diffraction efficiency of 72.8% is utilized to fabricate grating. The Femtosecond laser beam is accurately focused at the center axis of the SMF core using a cylindrical lens of a 25.5 mm focal length and two high precision three-axis stages. The grating growth process is monitored by an optical spectrum analysis (OSA) with a wavelength resolution of 0.02 nm. The FBG is formed with a length of 2 mm after 60 s laser exposure (i.e., 60,000 laser pulses), where the pulse energy is 6.5 mJ. The grating has excellent characteristics under high temperatures because it has the same principle with type-II damaged gratings [17]. Additionally, the grating quality depends on the laser focus position and power and irradiation time. Hence, the main challenge of this work is to successfully inscribe the high quality FBG by the femtosecond laser with a high-intensity, and avoiding damage to the FP cavity. The micrographs of the FBG and FP are shown in Figure 1b,c, respectively. Figure 1d depicts the reflection spectrum of the FP/FBG sensor monitored by OSA, in which the periodic interference fringe is generated by the FP cavity and the peak corresponds to the resonance wavelength of the FBG. The resonance wavelength of FBG is 1555.3 nm.

## 4. Experimental Results and Discussion

**Figure 3:**
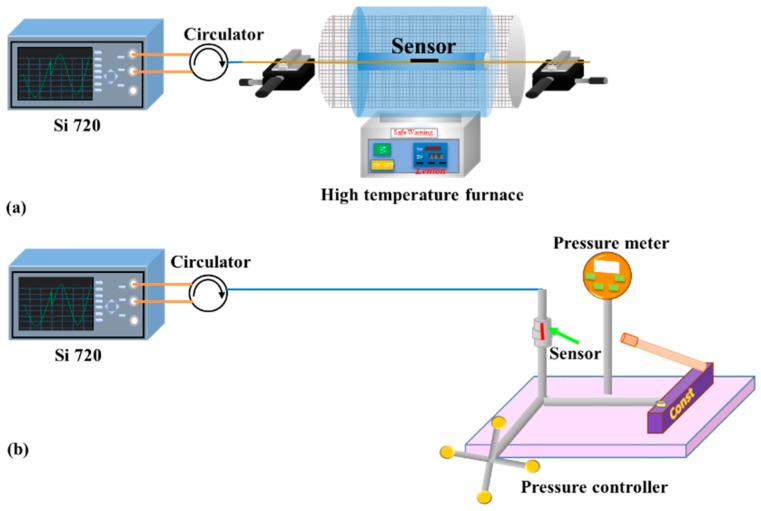
Schematic diagram of the experimental setup. (**a**) Strain and temperature tests; (**b**) pressure test at room temperature.

**Figure 4:**
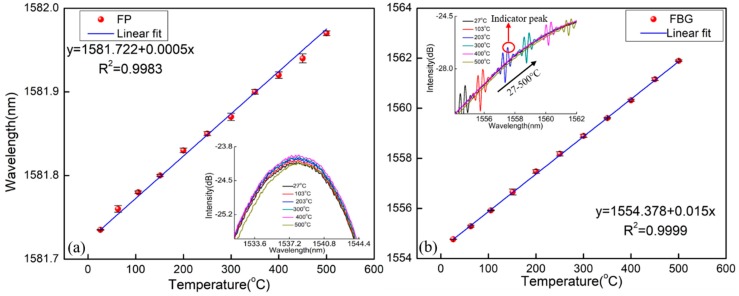
Temperature response of the sensor. (**a**) The FP; (**b**) the FBG.

Figure 3a shows the temperature experimental setup. The light from the sweeping laser source in Si720 was launched into the sensor head using a circulator. The optical spectrum analyzer (OSA) (Si720, Micron Optics, Atlanta, GA, USA) was used to monitor the reflection spectrum with a wavelength resolution of 2.5 pm and a wavelength scanning range of 1510~1590 nm. The temperature characteristic of the sensor was demonstrated from room temperature to 500 °C with a temperature interval of 50 °C, which was placed in a high temperature tube furnace with a temperature accuracy of ±1 °C (Lenton, Derbyshire, UK). The temperature of the furnace was kept constant for 20 min at each step to ensure uniform distribution of the temperature before each recording. The experiment was then repeated for three cycles to verify the repeatability of the sensor. The wavelengths of the FP and FBG were plotted as a function of the temperature, as shown in Figure 4. It is seen that the resonance wavelength has a red shift with the rising temperature and the temperature response has good repeatability. The temperature sensitivities of the FP and the FBG were 0.5 pm/°C and 15 pm/°C, respectively. In the insets of Figure 4, the reflection spectra of the sensor at different temperatures are plotted. The sensor has a good spectral profile at high temperatures and the FBG has two reflection peaks, in which the reflection peak at a long wavelength was the indicator peak and the peak at a short wavelength was the side lobe.

**Figure 5:**
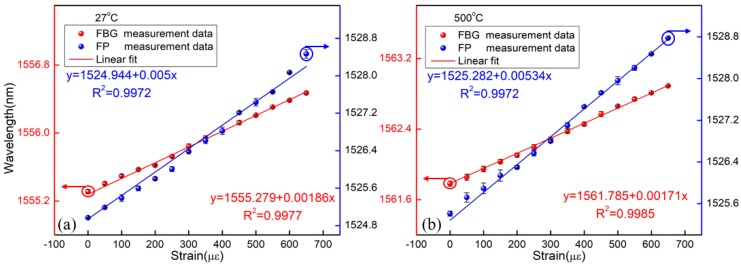
Strain responses of the sensor at different temperatures. (**a**) At 27 °C; (**b**) at 500 °C.

**Figure 6:**
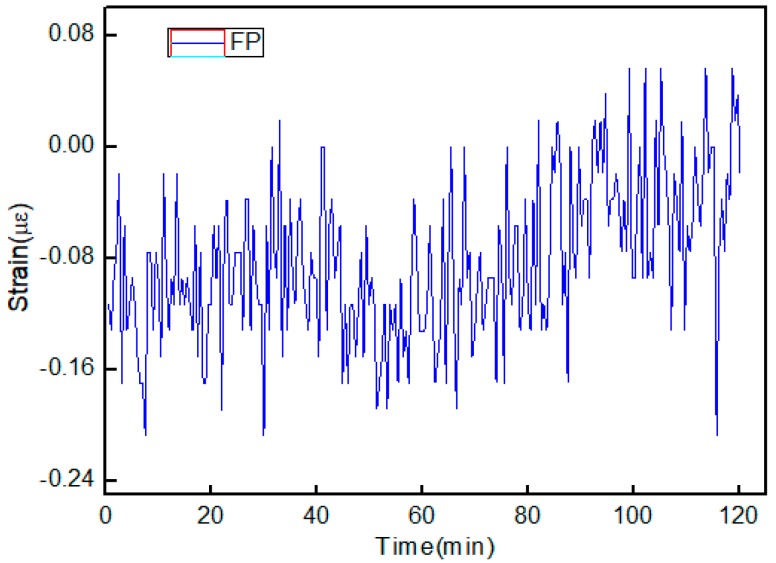
Stability of the FP strain sensor.

The strain responses of the FBG and FP cavity were tested at different temperatures, respectively. The sensor was placed in a tube furnace, and the experimental setup is shown in Figure 3a. Additionally, both sides were fixed on two one-dimensional stages with a displacement resolution of 0.01 mm. At each temperature point, the applied strains on the device were increased from 0 to 650 µε and then decreased down to 0 µε in steps of 50 µε. The applied strain was held for 10 min to achieve stable readings of the wavelengths at each step. Then, the experiment was conducted for three cycles to verify the repeatability of the sensor. During the whole tests, we recorded the two resonance wavelengths of the FBG and the FP, respectively. Figure 5 shows the resonance wavelength shift with the strain rising at two different temperatures. From this figure, it is seen that the strain sensitivities of the FP and the FBG were 5.0 pm/µε and 1.86 pm/µε at 27 °C, respectively. Simultaneously, the strain sensitivities of the FP and the FBG were 5.34 pm/µε and 1.71 pm/µε at 500 °C, respectively. Hence, the temperature and strain change could be obtained based on Equation (3) and Equation (4), respectively. For clarity, the strain sensitivities of the sensor at different temperatures are given in Table 1. It shows that the strain coefficients of the device were slightly different under different temperatures, which may result from the change of the properties of the silica material induced by heating. For the FP, the changes in the refractive index of the air in the FP cavity caused by the temperature also affected the strain sensitivity. Furthermore, at room temperature and standard atmospheric pressure, the strain stability of the FP sensor was obtained within 2 h when the sensor was fixed on the optical experimental platform, as shown in Figure 6. It was found that the fluctuation of the FP strain sensor was ±0.21 µε. This is mainly caused by the fluctuation of the light source and destabilization of the environment (temperature, vibration).

**Figure 7:**
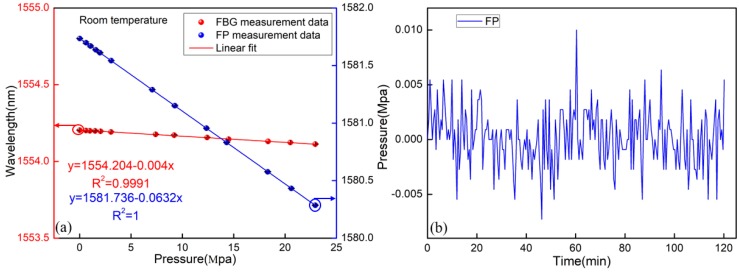
(**a**) Pressure responses of the sensor at room temperature; (**b**) stability of the FP pressure sensor.

The pressure characteristics of the FP and FBG were investigated at room temperature, respectively. The device was sealed in a metal tunnel. A pressure generator-controller was used to provide and control the pressure to the sensor. The calibration of the pressure applied to the sensor was achieved by the pressure meter with an accuracy of 0.05%, as shown in Figure 3b. The pressure was increased from 0 to 23 Mpa and then decreased down to 0 Mpa at an unequal interval. This process was carried out three times to verify the repeatability of the sensor. Additionally, the relationships between the wavelength and pressure are shown in Figure 7a. It is found that the pressure sensitivities of the FP and the FBG were −63.2 pm/MPa and −4 pm/MPa, respectively. The temperature and pressure variations could be obtained based on Equation (7) and Equation (8), respectively. The pressure stability of the FP was also tested within 2 h, as given by Figure 7b. It is found that the fluctuation of the FP pressure sensor was ±0.01 MPa. Similarly, it is mainly caused by the fluctuation of the light source and destabilization of the environment (temperature, vibration).

These experimental results prove the combined sensor is capable of achieving accurate measurement of strain/pressure and temperature. Moreover, as to the FP and fiber grating hybrid sensors, the proposed sensor has the potential advantages of an all-fiber sensor and high strain/pressure sensitivity, good thermal stability, and strong mechanical strength compared to those of capillary-based sensors [15,18] and previously reported sensors [9,11,15,19], such as FBG inscribed by ultraviolet laser [11,12] and RFBG [9,13], respectively, as shown in Table 2. Therefore, the proposed sensor has attractive sensing performances and huge potential for applications in harsh environments.

## 5. Conclusions

In this paper, a highly integrated all-fiber-optic sensor with a short FBG inscribed by an 800 nm femtosecond laser cascaded with an FP cavity fabricated by a 157 nm laser was proposed and demonstrated. The FBG is sensitive to temperature, but has a small strain/pressure sensitivity, while the FP cavity is sensitive to strain/pressure, but has a small temperature sensitivity. Additionally, the temperature sensitivity of the FP was 0.5 pm/°C. The sensor showed fairly good strain/pressure sensing performances over a specific temperature range. Additionally, the integrated sensor could be widely used in many fields for measurement of high temperature and strain/high-pressure due to the compact size of the all-fiber in-line sensor and the consistency of production.

## Figures and Tables

**Table 1:** Strain sensitivities of the sensor at different temperatures.

Temperature(°C)	Strain Sensitivity (pm/µε)	
	FP	FBG
27	5	1.86
100	5	1.77
200	5.32	1.88
300	5.14	1.62
400	5	1.63
500	5.34	1.71

**Table 2:** The performance of different combination structures of FP and fiber grating.

Sensor Structure	Grating Type	Strain/Pressure Sensitivity of the Interferometer	Reference
FP cascaded RFBG	RFBG	1.23 pm/µε (at 19 °C)	[9]
FBG cascaded a capillary-based FP	Inscribed by Femtosecond laser	1.74 pm/µε (at 23 °C)	[15]
Air cavity FP overlapped on RFBG	RFBG	3.3 pm/µε (at 50 °C)	[13]
Spheroidal-Cavity-Overlapped FBG	Inscribed by UV laser	3.76 pm/µε (at 25 °C)	[11]
PCF ^1^-Cavity FBG FP Resonator	Inscribed by UV laser	~10.1 pm/Mpa (at room temperature)	[19]
Air cavity FP cascaded FBG	Inscribed by Femtosecond laser	5 pm/µε, −63.2 pm/Mpa (at 27 °C)	In this work

^1^ Photonic crystal fiber (PCF).
